# Flux-Dependent Superconducting Diode Effect in an Aharonov–Bohm Interferometer

**DOI:** 10.3390/ma18204670

**Published:** 2025-10-11

**Authors:** Yu-Mei Gao, Hao-Yuan Yang, Feng Chi, Zi-Chuan Yi, Li-Ming Liu

**Affiliations:** 1 School of Electronic and Information Engineering, University of Electronic Science and Technology of China, Zhongshan Institute, Zhongshan 528400, China; yumeigao@zsc.edu.cn (Y.-M.G.); yizichuan@zsc.edu.cn (Z.-C.Y.); liulmxps@zsc.edu.cn (L.-M.L.); 2 South China Academy of Advanced Optoelectronics, South China Normal University, Guangzhou 510006, China; 2024024413@m.scnu.edu.cn

**Keywords:** supercurrent, superconducting diode effect, quantum dot, Aharonov–Bohm interferometer, magnetic flux

## Abstract

We theoretically investigate the supercurrent and superconducting diode effect (SDE) in an Aharonov–Bohm (AB) interferometer sandwiched between two aluminium-based superconducting leads. The interferometer features a quantum dot (QD), which is created in an indium arsenide (InAs) semiconductor nanowire by local electrostatic gating, inserted in one of its arms and a magnetic flux threading through the ring structure. The magnetic flux breaks the system time-reversal symmetry by modulating the quantum phase difference between electronic transport through the QD path and the direct arm, which enhances constructive interference in one direction and destructive interference in the other. This leads to a discrepancy between the magnitudes of the forward and reverse critical supercurrents and is the core mechanism that induces the SDE. We demonstrate that the critical supercurrents exhibit Fano line shapes arising from the interference between discrete Andreev bound states in the QD and continuous states in the direct arm. It is found that when electron transport is dominated by the QD-containing path as compared to the direct arm path of the interferometer, the diode efficiency reaches a maximum, with values as high as 80%. In contrast, when the direct arm path dominates transport, the diode efficiency becomes weak. This attenuation is attributed to the participation of higher-order quantum interference processes, which disrupt the nonreciprocal supercurrent balance. Importantly, the proposed AB interferometer system has a relatively simple structure, and the realization of the SDE within it is feasible using current nano-fabrication technologies.

## 1. Introduction

The superconducting diode effect (SDE), which refers to the difference in magnitude of critical supercurrents flowing in opposite directions, has been recently proposed and demonstrated in diverse superconducting systems [1,2,3,4,5,6,7]. In the conventional semiconductor p-n junction, imbalance of chemical potential distribution breaks system inversion symmetry and leads to nonreciprocal electric currents [8]. A semiconductor diode with resistance depending on the current flow direction is a kind of key component in electronic circuits. But the unavoidable heat generation by charge currents under external bias voltage in semiconductor diodes is a bottleneck problem impeding further integration of the circuit. On the contrary, the supercurrent is driven by the superconducting phase difference in Josephsoun junctions in the absence of bias voltage or resistance and thus there is no Joule heating [1,2,4,9]. The superconductor diode, then, should be a low-energy-consumption component in superconducting devices for quantum computing and communication [4,10,11,12]. From the basic research aspect, the SDE is a powerful means to study diverse topics in condensed matter physics, for example the connections between symmetry [13], spin–orbit coupling [14,15,16,17], vortex dynamics [18], and unconventional superconductors [19]. Until now, the SDE has been demonstrated in various non-centrosymmetric superlattices [5], Josephson junctions [20], nano-fabricated devices [21], and thin films under a magnetic field [22].

In general, the SDE originates from breaking of either inversion or time-reversal symmetry, or both [2,3,4,10]. The inversion symmetry can be broken by the asymmetry of a stacked heterostructure [5], an artificial device’s interface [6] or edge [23], and a crystal lattice [7]. The time-reversal asymmetry usually is achievable with the help of external magnetic fields [9] or mechanism relating to the nature of the magnetic layers [24], valley polarization [25], interface magnetism [6,26], etc. In multi-terminal or multi-path superconducting systems, the magnetic field generates a magnetic flux, which will lead to the time-reversal symmetry being broken and induce a nonreciprocal supercurrent through the superconducting quantum interference effect [27,28,29,30,31]. For example, a four-terminal double-loop superconducting quantum interference device (SQUID) was recently realized in an indium arsenide/aluminum two-dimensional heterostructure [32]. A remarkable SDE characterized by a diode efficiency as large as 34% was observed with the help of magnetic fluxes and gate voltages. Subsequently, a simplified two-terminal double-loop SQUID inserted between Josephson junctions was studied [33,34]. A nonreciprocal critical supercurrent emerges due to the lack of both inversion and time-reversal symmetries by threading two magnetic fluxes individually through the two loops.

In the last two decades, interferometers coupled to superconductor leads with semiconductor quantum dots (QDs) inserted in their arms have received extensive attention [27,35,36,37,38,39,40,41,42,43]. Since the size of QDs is in the nanometer scale in two of the three directions, the confined electron exhibits a discrete electronic state and fully adjustable quantum levels [44,45]. These unique properties make semiconductor QDs have extremely broad applications in single-electron devices, memory devices, and various optical devices. The confined electron in hybridized superconductor/QD devices can couple to Cooper pairs in the superconductors and form Andreev bound states (ABSs) in the superconducting gap, becoming the carrier of the supercurrent [27,35,36,37,38,39,40,41,42,43,46,47,48,49]. Recently, an AB interferometer with two QDs on each of its arms was sandwiched between two superconductors in experiments [40,41,42]. The amplitude, transport directions, and period with respect to the phase difference of the supercurrent in these devices can be efficiently varied by the combined actions of the ABSs, magnetic flux, and the spin–orbit interactions (SOIs) of Rashba type on the QDs. Besides the phase difference between the superconductor leads, the phase arisen from the SOI may also serve as a driving force for the supercurrent and results in the SDE [43]. A significant SDE in an AB interferometer with a QD inserted in one of its two arms was also proposed theoretically very recently [16]. The working mechanism relies on the Zeeman splitting from a vertically applied magnetic field and the SOI-induced phase. With the help of gate voltages that adjust the QD’s energy levels, a large value of diode efficiency reaching up to 70% can be achieved.

**Figure 1:**
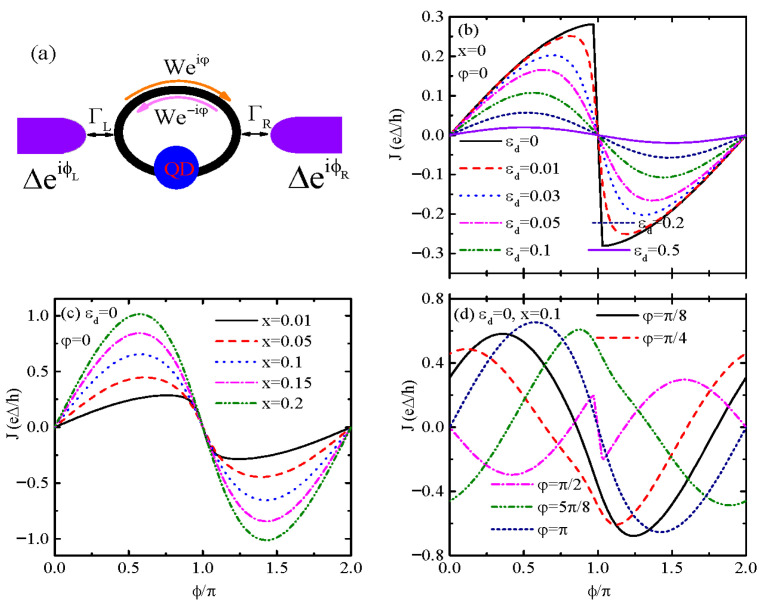
(**a**) Schematic plot of the device with an Aharonov–Bohm (AB) interferometer, in which a quantum dot (QD) is situated in one arm and a magnetic flux φ penetrates through the ring. The two superconductor leads with an energy gap Δ and phases $\phi_{L/R}$ couple to the QD with strengths of $\Gamma_{L/R}$ and to each other through a tunnel barrier with strength $We^{i\varphi }$. It is noted that due to the applied magnetic flux, which breaks the system time-reversal symmetry, a phase factor φ is added in *W* and then the forward tunnel amplitude $We^{i\varphi }$ is different from the backward tunnel one $We^{-i\varphi }$. (**b**–**d**) Show the Josephson current *J* as a function of the phase difference ϕ for the indicated parameters. In the QD–ring structure (x≠0), the magnitudes of the forward and backward Josephson currents become different from each other due to the existence of magnetic flux φ and induce a diode effect as in (**d**).

Here we propose a system for the SDE in Figure 1a. It consists of an AB interferometer sandwiched between two superconductor leads. The supercurrent driven by the two superconductors’ phase difference becomes nonreciprocal due to a magnetic flux penetrating through the ring and by properly changing the QD’s energy level. As compared to previous similar setups [16,43], both the configuration and working mechanism of the present one are quite simple, as it requires a single-level QD and magnetic flux only. The AB effect predicts that the phase of the wavefunction of electrons flowing in a metal ring is affected by an externally applied magnetic flux that passes through the ring, even if the electrons do not directly experience the magnetic field *per se* [50]. This theoretical prediction of Aharonov and Bohm is based on the fact that the magnetic vector potential, rather than the magnetic field, is the underlying fundamental physical entity from a quantum mechanical point of view. Now, this prediction has been clarified experimentally. While originally the AB effect referred to zero magnetic field experienced by the electrons, later it was clarified that this was not a necessity [28,30,31,32,33]. Our results indicate that when the transport processes are dominated by the the QD path as compared to the direct arm path, the maximum value of the diode efficiency can be enhanced up to 80%. But if the direct arm path dominates, the diode efficiency value becomes small.

Beyond the fundamental exploration of quantum interference and nonreciprocal supercurrent dynamics, the AB interferometer-based superconducting diode proposed in this work exhibits substantial potential for practical applications in advanced superconducting electronics [10]. First, in the field of quantum computing, where low energy consumption and minimal decoherence are critical, this diode’s zero-Joule-heating supercurrent transport and high tunability via QD energy levels or magnetic flux make it an ideal candidate for on-chip signal rectification and quantum bit control [10,51]. It can efficiently filter out reverse supercurrent noise that would otherwise disrupt qubit coherence, while its high maximum diode efficiency ensures minimal energy loss, which is a key advantage over existing SDE devices with lower efficiency [10,11,51]. Second, in superconducting microwave circuits for quantum communication [31], the diode’s simple structure, compatible with current nano-fabrication, enables monolithic integration with other superconducting components, facilitating the development of compact, high-performance signal processing modules such as superconducting rectifiers or isolators [12]. Finally, in low-temperature electronic systems such as cryogenic sensors, the diode’s ability to operate without resistive heating reduces the load on cryocoolers, extending system lifetime and lowering operational costs [10,11]. These applications not only leverage the unique strengths of the proposed system but also address critical bottlenecks in current superconducting technology, paving the way for more efficient and integrated quantum and low-temperature electronic devices.

## 2. Model and Method

The present device, with two superconductor leads that couple to a QD and simultaneously to each other through a tunnel barrier serving as the direct arm, is shown in Figure 1a. Here we consider a single-level QD, and the system Hamiltonian is modeled by [16,21,35,36,37],

(1) $$\begin{array}{cc} H & =\sum_{\sigma }\varepsilon_{d}d_{\sigma }^{†}d_{\sigma }+\sum_{\alpha ,k,\sigma }\varepsilon_{\alpha ,k\sigma }C_{\alpha ,k\sigma }^{†}C_{\alpha ,k\sigma }+\sum_{\alpha ,k}\left(\Delta_{\alpha }e^{i\phi_{\alpha }}C_{\alpha ,k↑}^{†}C_{\alpha ,-k↓}+H.c.\right)\\ & +\sum_{\alpha ,k,\sigma }\left(V_{\alpha }C_{\alpha ,k\sigma }^{†}d_{\sigma }+H.c.\right)+\sum_{k,k^{\prime },\sigma }\left(W^{-i\varphi }C_{L,k\sigma }^{†}C_{R,k^{\prime }\sigma }+H.c.\right), \end{array}$$

in which $d_{\sigma }^{†}$$\left(d_{\sigma }\right)$ creates (annihilates) an electron in the QD with energy level $\varepsilon_{d}$ and spin direction σ=↑,↓. The operator $C_{\alpha ,k\sigma }^{†}\left(C_{\alpha ,k\sigma }\right)$ denotes creation (annihilation) of electrons in the superconductor lead α(α=L,R) with an energy of $\varepsilon_{\alpha ,k\sigma }$, energy gap $\Delta_{\alpha }$, and superconducting phase $\phi_{\alpha }$. Here we study the supercurrent arisen from the phase difference $\phi_{L}-\phi_{R}$ in the absence of bias voltage. In addition, chemical potentials of the left and right leads are set to be $\mu_{L}=\mu_{R}=0$. The quantity $V_{\alpha }$ in the forth term of Hamiltonian (1) describes the hopping amplitude between QD and lead-α. The last term in Hamiltonian (1) is for the direct coupling between the left and right superconductor leadss, with a hopping amplitude of *W*. Due to the applied vertical magnetic field with strength *B*, a phase factor φ is added in *W* with $\varphi =\int \vec{A}\cdot d\vec{r}/\varphi_{0}$, in which $\vec{A}=\left(-By,0,0\right)$ and $\varphi_{0}=\hbar /e$ are individually the vector potential and flux quanta [35,36,37].

The Josephson supercurrent *J* through the QD-AB ring is obtained from the time evolution of the particle number operator of electrons $N_{L}=\sum_{k,\sigma }C_{L,K\sigma }^{†}C_{L,k\sigma }$ in the left superconductor lead [35,36,37,47,48]:

(2) J=−e<dNLdt>=4eℏ∫dε2πRe[VLGdL,11<(ε)+We−iφGRL,11<(ε)],

in which Green’s functions $G_{dL}^{<}\left(\varepsilon \right)$ and $G_{RL}^{<}\left(\varepsilon \right)$ are individually the Fourier transformation of $G_{dL}^{<}\left(t,t\right)$ and $G_{RL}^{<}\left(t,t\right)$. In the Nambu representation, they are given by [21,37],

(3) $$\begin{array}{c} G_{dL}^{<}\left(\varepsilon \right)=i\sum_{k}\left[\begin{array}{cc} \;\;<C_{L,k↑}^{†}\left(t\right)d_{↑}\left(t\right)> & <C_{L,k↑}^{†}\left(t\right)d_{↓}^{†}\left(t\right)>\\ \;\;<C_{L,-k↓}\left(t\right)d_{↑}\left(t\right)> & <C_{L,-k↓}\left(t\right)d_{↓}^{†}\left(t\right)> \end{array}\right], \end{array}$$

and

(4) $$\begin{array}{c} G_{dL}^{<}\left(\varepsilon \right)=i\sum_{k,k^{\prime }}\left[\begin{array}{ccc} \;\;<C_{L,k↑}^{†}\left(t\right)C_{R,k^{\prime }↑}\left(t\right)> & & <C_{L,k↑}^{†}\left(t\right)C_{R,-k^{\prime }↓}^{†}\left(t\right)>\\ \;\;<C_{L,-k↓}\left(t\right)C_{R,k^{\prime }↑}\left(t\right)> & & <C_{L,-k↓}\left(t\right)C_{R,-k^{\prime }↓}^{†}\left(t\right)> \end{array}\right]. \end{array}$$

Notice that the Josephson current arises from the superconductors’ phase difference without bias voltage, and then the device is in equilibrium and Green’s function obeys the fluctuation–dissipation theorem [21,37] $G^{<}\left(\varepsilon \right)=-f\left(\varepsilon \right)\left[G^{r}\left(\varepsilon \right)-G^{a}\left(\varepsilon \right)\right]$, where $f\left(\varepsilon \right)=1/\left[1+e^{\varepsilon /k_{B}T}\right]$ is the equilibrium Fermi distribution function and $k_{B}$ and *T* are individually the Boltzmann constant and system temperature *T*. $G^{r/a}\left(\varepsilon \right)$ is the retarded/advanced Green function and $G^{a}\left(\varepsilon \right)={\left[G^{r}\left(\varepsilon \right)\right]}^{†}$. We next calculate the retarded Green function $G^{r}\left(\varepsilon \right)$ with the help of the Dyson equation technique [21,37]

(5) $$\begin{array}{c} G^{r}\left(\varepsilon \right)=g^{r}\left(\varepsilon \right)+g^{r}\left(\varepsilon \right)\Sigma^{r}\left(\varepsilon \right)G^{r}\left(\varepsilon \right), \end{array}$$

in which $G^{r}\left(\varepsilon \right)$ is defined in a space composed of three regions: the left and right superconductor leads and the QD,

(6) $$\begin{array}{c} G^{r}\left(\varepsilon \right)=\left[\begin{array}{ccc} \;\;G_{LL}^{r}\left(\varepsilon \right) & G_{LR}^{r}\left(\varepsilon \right) & G_{LD}^{r}\left(\varepsilon \right)\\ \;\;G_{RL}^{r}\left(\varepsilon \right) & G_{RR}^{r}\left(\varepsilon \right) & G_{RD}^{r}\left(\varepsilon \right)\\ \;\;G_{DL}^{r}\left(\varepsilon \right) & G_{DR}^{r}\left(\varepsilon \right) & G_{DD}^{r}\left(\varepsilon \right) \end{array}\right]. \end{array}$$

The self-energy $\Sigma^{r}$ in Equation (5) is defined similarly as

(7) $$\begin{array}{c} \Sigma^{r}=\left[\begin{array}{ccc} \;\;0 & \Sigma_{LR}^{r} & \Sigma_{L}^{r}\\ \;\;\Sigma_{LR}^{r†} & 0 & \Sigma_{R}^{r}\\ \;\;\Sigma_{L}^{r†} & \Sigma_{R}^{r†} & 0 \end{array}\right], \end{array}$$

in which the diagonal 2×2 matrices $\Sigma_{L/R}^{r}$ and $\Sigma_{LR}^{r}$ represent coupling between the left/right superconductor with the QD and with each other, respectively. They are given individually by $\Sigma_{L/R}^{r}=\mathrm{diag}\left(V_{L/R},-V_{L/R}\right)$ and $\Sigma_{LR}^{r}=\mathrm{diag}\left(We^{i\varphi },-We^{-i\varphi }\right)$. $g^{r}\left(\varepsilon \right)$ in Equation (5) stands for the free retarded Green function of the system without coupling between the leads and the QD with $g^{r}\left(\varepsilon \right)=\mathrm{diag}\left[g_{LL}^{r}\left(\varepsilon \right),g_{RR}^{r}\left(\varepsilon \right),g_{DD}^{r}\left(\varepsilon \right)\right]$. Green’s function of the isolated leads is written as [16,21]

(8) $$\begin{array}{c} g_{\alpha \alpha }^{r}=-i\pi \rho_{\alpha }\left(\varepsilon \right)\gamma_{\alpha }\left(\varepsilon \right)\left[\begin{array}{cc} \;\;1 & -\frac{\Delta_{\alpha }}{\varepsilon }e^{-i\phi_{\alpha }}\\ \;\;-\frac{\Delta_{\alpha }}{\varepsilon }e^{i\phi_{\alpha }} & 1 \end{array}\right], \end{array}$$

where $\rho_{\alpha }\left(\varepsilon \right)$ is the normal density of states of lead-α. The quantity $\gamma_{\alpha }\left(\varepsilon \right)$ is given by [21,47]

(9) $$\begin{array}{c} \gamma_{\alpha }\left(\varepsilon \right)=\frac{\left|\varepsilon |\vartheta (|\varepsilon |-\Delta_{\alpha }\right)}{\sqrt{\varepsilon^{2}-\Delta_{\alpha }^{2}}}+\frac{\varepsilon \vartheta (\Delta_{\alpha }-|\varepsilon |)}{i\sqrt{\Delta_{\alpha }^{2}-\varepsilon^{2}}}, \end{array}$$

in which ϑ(x)=1 when x>0 and ϑ(x)=0 otherwise. For an isolated QD, Green’s function is solved as $g_{DD}^{r}\left(\varepsilon \right)=\mathrm{diag}\left[1/\left(\varepsilon -\varepsilon_{d}+i\delta \right),1/\left(\varepsilon +\varepsilon_{d}+i\delta \right)\right]$ [16,21].

To study the SDE effect, we introduce the diode efficiency given by [21]

(10) $$\begin{array}{c} \eta =\frac{J_{c+}-\left|J_{c-}\right|}{(J_{c+}+|J_{c-}|)/2}, \end{array}$$

in which $J_{c+}$ ($J_{c-}$) is the positive (negative) critical current, which is obtained by choosing the maximum (minimum) Josephson current in a 2π period of the superconductor phase [16,21,46,51]. As a key parameter distinguishing superconductors from ordinary conductors, the experimentally measurable critical current determines the stability of superconducting devices and systems during operation [51].

## 3. Numerical Results

During numerical calculations, we focus on the zero-temperature condition (T=0) and assume identical supreconducting gaps of the two leads ($\Delta_{L}=\Delta_{R}=\Delta$), in which Δ≡1 is set to be the energy unit [16,21,37]. It is further assumed that the central region composed of the QD and the direct arm is connected to the leads with the same strengths described by the line-width function $\Gamma =2\pi \rho_{\alpha }V_{\alpha }^{2}$ and $x={\left(\pi \rho_{\alpha }W\right)}^{2}$, respectively. Figure 1b–d show the Josephson current *J* varying with respect to the superconductors’ phase difference $\phi =\phi_{L}/2=-\phi_{R}/2$. When the direct arm is disabled (x=0), as shown in Figure 1b, the present system reduces to a QD sandwiched between two superconductors (S-QD-S), and the J−ϕ curve obeys the relation of J(ϕ)=−J(ϕ+π), which is a signature of symmetric ABS contributions. If the QD’s energy level $\varepsilon_{d}$ aligns with the Fermi energy of the leads $E_{F}=0$, the J−ϕ curve shows an approximately triangular line shape and jumps from positive to negative at ϕ=π. This is because the ABSs degenerate at the Fermi energy [46] and eliminate nonreciprocity (no SDE). When the QD’s level is shifted away from the Fermi energy ($\varepsilon_{d}\neq 0$), the J−ϕ is usually a sinuous line shape. Now the supercurrent is suppressed by increasing $\varepsilon_{d}$. The reason is that now the ABSs depart from the Fermi energy and break down the resonance, leading to the suppression of the current [37,46,47,48]. As shown in Figure 1c, when the two superconductor leads are directly coupled (x≠0) for $\varepsilon_{d}=0$, the amplitude of *J* is obviously enhanced due to the additional electron transport channel. Now the J−ϕ curve becomes a sinuous shape with retained J(ϕ)=−J(ϕ+π) property without magnetic flux as the time-reversal symmetry is preserved [37,46]. In the presence of magnetic flux φ≠0, Figure 1d shows that the Josephson current is strongly varied, and now the current–phase curve does not preserve the sinusoidal-like shape [37]. The current can become non-zero at ϕ=nπ with integer n=0,±1,±2,⋯⋯. Moreover, the maxima of the forward and reverse currents can be different, or in other words, the magnitudes of the positive and negative critical currents are not equal ($J_{c+}\neq \left|J_{c-}\right|$). The nonreciprocity of the supercurrent arises because φ modulates the phase difference between electrons traversing the QD and direct arm, creating constructive interference in one direction and destructive in the other. Figure 1 confirms that the magnetic flux φ is mandatory for breaking symmetry and inducing the SDE.

**Figure 2:**
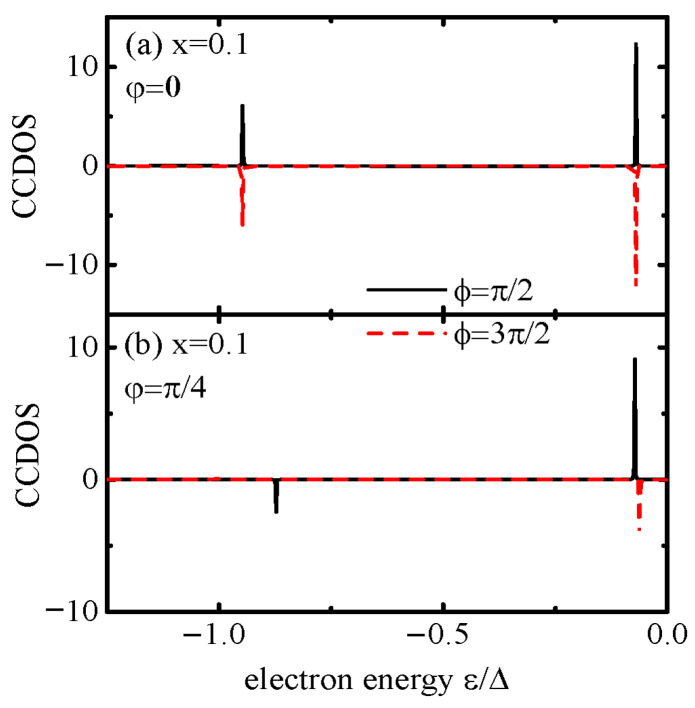
Current-carrying density of states (CCDOS) as a function of energy ε for φ=0 in (**a**) and φ=π/2 in (**b**), respectively. Other parameters are line-width function Γ=0.1 and dot level $\varepsilon_{d}=0$.

To fully understand the nonreciprocal Josephson current, we plot the current-carrying density of states (CCDOS) j(ε)=Re[VLGdL,11<(ε)+We−iφGRL,11<(ε)] [21,48], which quantifies how electronic states contribute to the supercurrent and directly links ABS behavior to nonreciprocal current for the cases with and without the magnetic flux in Figure 2a and Figure 2b, respectively. As is well known, the supercurrent *J* has two parts; one is the continuous part due to electrons with energy ε outside the energy gap of the superconductors, whereas the other is a discrete one from electrons with energy within the gap [46,47,48]. The continuous part can be calculated directly by the integral of the CCDOS, while the discrete part is approached by solving poles of the denominator of the CCDOS, which are the ABSs. Generally, the supercurrent amplitude arisen from the discrete spectrum will be stronger than that due to the continuous spectrum. Moreover, the ABSs are paired with energy of opposite signs. Under the condition of zero temperature, only those with an energy of [−Δ,0] relate to the current. Affected by direct connection of the two leads, instead of one bound state in a S-QD-S junction in the region of [−Δ,0], there are two bound states arisen from hybridization of the QD $E_{QD}^{-}$ and the arm $E_{A}^{-}$ [37]. The latter depends on the magnetic flux through $E_{A}^{-}=-\Delta \sqrt{1-\frac{4x}{{\left(1+x\right)}^{2}}\sin^{2}\left(\varphi /2\right)}$. At φ=0, as shown in Figure 2a, the CCDOS exhibits a symmetric δ-like peak due to the ABS satisfying the relation of $E^{-}\left(\phi /2\right)=-E^{-}\left(3\phi /2\right)$. This symmetry ensures that the positive and negative cancel out $|J_{c+}\left|=\right|J_{c-}|$, as the ABS contribution to current is identical in both directions. In the presence of magnetic flux (φ=π/4, for example) that disrupts ABS symmetry, Figure 2b shows that the bound state near Δ is shifted from a positive value to a negative one, whereas that near $E_{F}=0$ remains almost unchanged for ϕ=π/2, leading to an obviously reduced positive current. While for ϕ=3π/2, the original bound state near Δ is suppressed to zero, that near $E_{F}$ survives and the negative current will be slightly reduced as compared to the case of the positive current, leading to a nonreciprocal current and the SDE.

**Figure 3:**
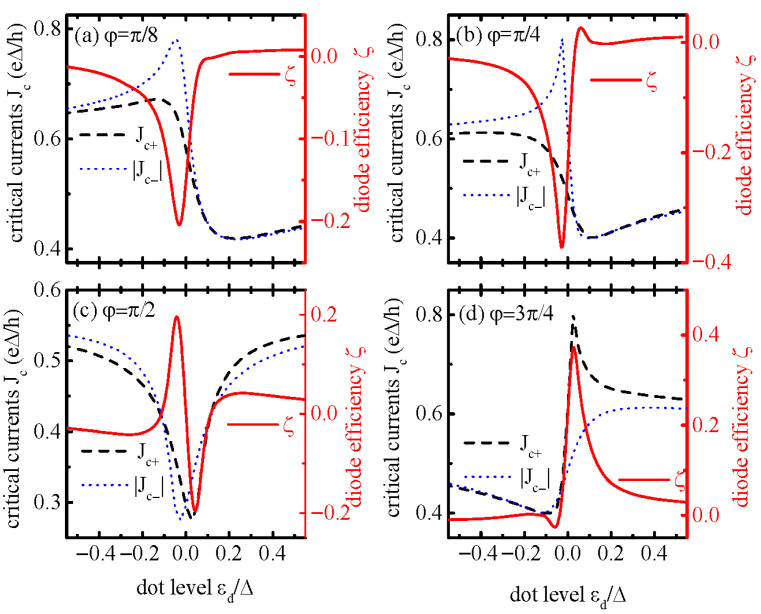
Positive critical Josephson current $J_{c+}$, absolute value of the negative Josephson current $|J_{c-}|$, and the diode efficiency ζ as functions of the dot level $\varepsilon_{d}$ for φ=π/8 in (**a**), φ=π/4 in (**b**), φ=π/2 in (**c**), and φ=3π/4 in (**d**). Other parameters are x=Γ=0.1.

We then study the diode efficiency ζ and critical currents $J_{c+}\left(J_{c-}\right)$ through the system in Figure 3. For the case of magnetic flux phase φ=nπ (n=0,±1,±2,⋯⋯), the current *J* is antisymmetric with respect to φ=nπ and $J_{c+}=\left|J_{c-}\right|$, leading to zero diode efficiency (ζ=0). Apart from φ=nπ, the Josephson current is nonreciprocal ($J_{c+}\neq \left|J_{c-}\right|$) and induces the SDE, which is presented in Figure 3. For φ=π/8 or π/4, as individually shown in Figure 3a,b, the extinct SDE characterized by negative ζ occurs in dot level regimes of $\varepsilon_{d}<0$. In the case of magnetic flux phase factor φ=π/2, ζ becomes antisymmetric due to the electron–hole symmetry given in Figure 3c. Increasing the value of φ>π/2, large positive diode efficiency emerges in the positive dot level regime, which is indicated in Figure 3d. It is worth noting that, due to the existence of a direct arm between the two leads, the critical current $J_{c\pm }$ as a function of the energy level of the QD $\varepsilon_{d}$ exhibits asymmetric peak–dip features, which are hallmarks of Fano resonance. This arises from interference between discrete ABS states in the QD and continuous states in the direct arm [37]. In the absence of magnetic flux (φ=0) and small *x*, the Fano resonant peak emerges at $\varepsilon_{d}=-\Gamma \sqrt{x}/\left(1-x\right)$, and the dip at approximately $\varepsilon_{d}=\Gamma /\left(2\sqrt{x}\right)$. Correspondingly, the diode efficiency ζ versus QD level also exhibits a Fano line-shape, which holds true in the presence of magnetic flux. This tunability is critical for experimenters; i.e., by adjusting $\varepsilon_{d}$ to align with Fano peaks, one can optimize ζ without modifying the device’s physical structure.

**Figure 4:**
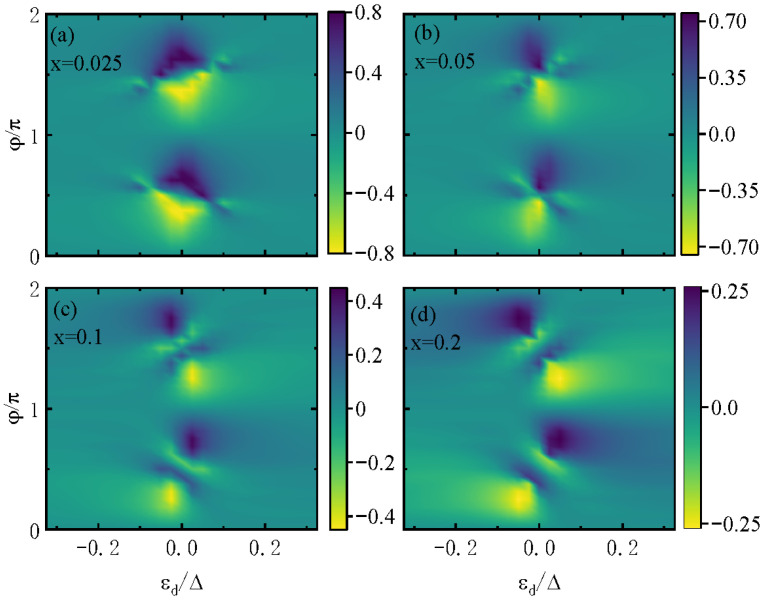
Counter plot of the diode efficiency ζ varying with respect to the dot level $\varepsilon_{d}$ and magnetic flux φ for direct coupling strength x=0.025 in (**a**), x=0.05 in (**b**), x=0.1 in (**c**), and x=0.2 in (**d**). The line-width function is fixed at Γ=0.1.

Figure 4 presents a counter plot of the diode efficiency varying with the QD’s level and the flux φ. Several characteristics are worth noting: (1) The value of the diode efficiency can reach as large as ±0.8 for small x=0.025 in a relatively wide regime of $\varepsilon_{d}$ and φ (Figure 4a) and then decreases with increasing *x*, as in Figure 4b–d. In previous theoretical work, the diode efficiency could reach as high as 70% in the presence of a magnetic field and spin–orbit interaction in a similar AB-QD ring structure [16]. When the QD is coupled to a magnetic impurity, the diode efficiency is about 40%, which is the typical value found in experiments [30,31,32]. The broad high-efficiency region in Figure 4a (x=0.025) is ideal for experiments, as it tolerates small variations in $\varepsilon_{d}$ and φ, which are common in fabrication, without significant efficiency loss. (2) For a small *x* value, ζ changes in a relative small regime of φ because the direct path is almost closed, as shown in Figure 4a. With increasing *x*, the oscillation of ζ−φ is strong, as given in Figure 4b–d. The oscillation shape obviously departs from the sinφ or cosφ shape [37]. This is because larger *x* strengthens the direct arm, introducing higher-order tunneling processes that disrupt ABS symmetry and reduce nonreciprocity. (3) The diode efficiency obeys the relation of $\zeta \left(\varepsilon_{d},\varphi \right)=\zeta \left(-\varepsilon_{d},\varphi +\pi \right)$ because of the electron–hole symmetry and $J_{c}\left(\varepsilon_{d},\varphi \right)=J_{c}\left(-\varepsilon_{d},\varphi +\pi \right)$. ζ is a periodic function of magnetic flux φ of period π when $\varepsilon_{d}=0$ and period 2π for the case of $\varepsilon_{d}\neq 0$. This result is the same as that of the critical current, which was found in Ref. [37]. This periodicity shift is a unique signature of the AB interferometer: φ modulates the quantum phase of the direct arm, and $\varepsilon_{d}$ breaks electron–hole symmetry to alter the period.

**Figure 5:**
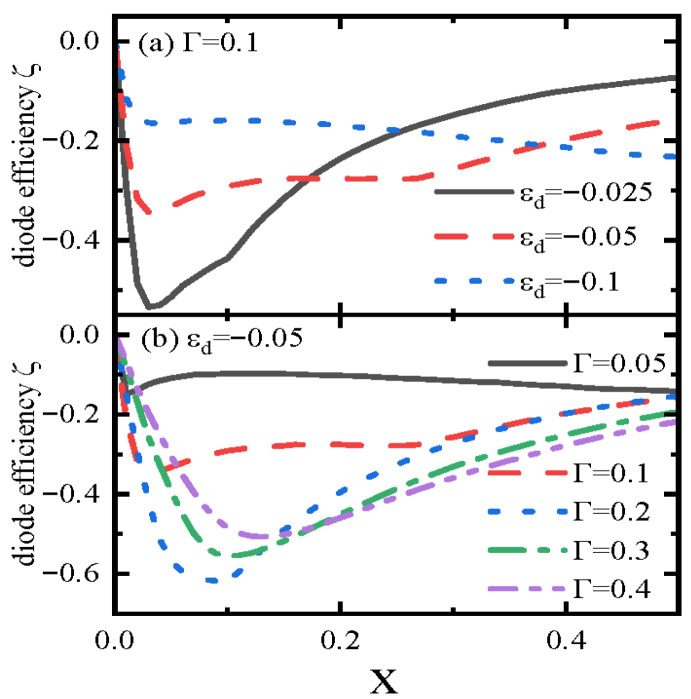
Diode efficiency ζ versus x for different values of $\varepsilon_{d}$ in (**a**) and different Γ in (**b**). The magnetic flux is set to be φ=π/4.

We then examine the impacts of direct coupling strength between the leads *x* on the diode efficiency for different QD levels $\varepsilon_{d}$ in Figure 5a and line-width functions Γ in Figure 5b. Since the diode efficiency satisfies the relation of $\zeta \left(\varepsilon_{d},\varphi \right)=\zeta \left(-\varepsilon_{d},\varphi +\pi \right)$, we only present the cases of negative QD levels $\varepsilon_{d}<0$ and φ=π/4. As shown in Figure 5a, ζ is negative for the chosen parameters. For all $\varepsilon_{d}<0$, the absolute value of ζ first increases with *x* up to about 0.05, reaches a maximum, and then decreases as *x* exceeds Γ=0.1. This non-monotonic trend reflects the “path dominance transition”; i.e., when x<Γ the QD path dominates the transport processes as compared with the direct arm path, and ζ is large because increasing *x* adds a weak direct arm that enhances quantum interference with the QD path and strengthens ABS asymmetry. When x>Γ, where the direct arm path dominates the transport, the absolute value of the diode efficiency is relatively small and depends nonlinearly on the value of *x*, as higher-order tunneling processes such as multiple electron scattering between leads reduce ABS contributions and weaken ζ. These results are confirmed by Figure 5b, where the diode efficiency shows similar behaviors to those in Figure 5a. For larger Γ (e.g., Γ=0.3), the maximum ζ shifts to larger x≈0.15, as a stronger QD–superconductor coupling delays the transition to direct path dominance. This confirms that Γ can be tuned to extend the high-efficiency regime to larger *x*, which is useful for balancing efficiency with current-carrying capacity.

The results of this work demonstrate that AB interferometers with a single QD are a promising platform for a high-efficiency SDE, with efficiency reaching up to 80% driven by QD-dominated transport and flux-tuned quantum interference. However, translating this performance to practical devices requires addressing several challenges. The first challenge is the stability and uniformity of magnetic flux because the SDE in this system relies on precise control of φ, including magnetic field inhomogeneity, which causes non-uniform φ across the ring, magnetic fields from nearby superconductors or qubits, or flux creep due to slow magnetic flux dissipation over time. This problem may be overcome by fabricating on-chip microcoils directly above the AB ring to generate localized, uniform magnetic fields and using flux-locked loops with SQUID sensors to stabilize φ or designing the AB ring with a symmetric, compact geometry to reduce flux inhomogeneity and edge effects [30,32,33]. The second challenge is the precise tuning of $\varepsilon_{d}$ to align with the Fermi energy of superconducting leads. However, in experimental implementations, QDs are susceptible to charge noise from trapped charges in the substrate or gate dielectrics and thermal fluctuations, which cause $\varepsilon_{d}$ to drift over time. This drift shifts the QD away from resonance with ABSs, reducing nonreciprocity and efficiency. This maybe resolved by employing coupled QD systems such as double QDs to create more robust resonance states that are less sensitive to local noise [41,42] or using top-gate architectures with high-k dielectrics to minimize charge trapping and improve gate capacitance, enabling finer and more stable $\varepsilon_{d}$ control [45,46]. The third challenge is the temperature sensitivity of ABSs [46]. The present theoretical model assumes zero temperature, where only ABSs in the energy range [−Δ,0] contribute to the supercurrent. At non-zero temperatures, thermal excitation generates quasi-particles that scatter ABSs, reducing their contribution to the nonreciprocal current [7,46]. Additionally, higher temperatures widen the thermal distribution of electrons, blurring the distinction between positive and negative critical currents. Potential solutions include the usage of high-critical-temperature superconductors as leads, which operate at higher temperatures while maintaining large superconducting gaps, reducing quasi-particle excitation [7].

## 4. Conclusions

In conclusion, this study presents a theoretical analysis of the Josephson supercurrent and the associated superconducting diode effect (SDE) in an Aharonov–Bohm (AB) interferometer, which is constructed by connecting a QD-containing arm and a direct arm to two superconducting leads. Our key results reveal that both the magnitude of the supercurrent and its oscillation period with respect to the superconducting phase difference between the two leads are strongly dependent on two critical factors: the energy level of the QD $\varepsilon_{d}$ and the magnetic flux φ penetrating the AB interferometer ring. The quantum interference effect, driven by these factors, generates a nonreciprocal critical supercurrent, which is an experimentally measurable phenomenon that directly gives rise to the SDE. We find that the diode efficiency ζ adheres to specific rules: It satisfies the relation of $\zeta \left(\varepsilon_{d},\varphi \right)=\zeta \left(-\varepsilon_{d},\varphi +\pi \right)$ due to electron–hole symmetry and the intrinsic characteristics of the critical supercurrent. Additionally, the diode efficiency exhibits periodicity with respect to the magnetic flux: it has a period of π when the QD energy level $\varepsilon_{d}=0$ and a period of 2π if $\varepsilon_{d}\neq 0$. A critical practical insight from this work is the achievement of high diode efficiency by optimizing three core parameters, i.e., the direct coupling strength between the superconducting leads, the energy level of the QD, and the magnetic flux through the ring. Specifically, maximizing the diode efficiency requires ensuring that electron transport is dominated by the QD-containing path rather than the direct arm path.

## Data Availability

The original contributions presented in this study are included in the article. Further inquiries can be directed to the corresponding author.
